# Acoustic Waves in Piezoelectric Layered Structure for Selective Detection of Liquid Viscosity

**DOI:** 10.3390/s23177329

**Published:** 2023-08-22

**Authors:** Andrey Smirnov, Vladimir Anisimkin, Elizaveta Shamsutdinova, Maria-Assunta Signore, Luca Francioso, Kirill Zykov, Vladimir Baklaushev, Iren Kuznetsova

**Affiliations:** 1 Kotel’nikov Institute of Radio Engineering and Electronics of RAS, Moscow 125009, Russia; anis@cplire.ru (V.A.); shes1996@bk.ru (E.S.); kuziren@yandex.ru (I.K.); 2 National Research Council of Italy, Institute for Microelectronics and Microsystems, 73100 Lecce, Italy; mariaassunta.signore@cnr.it (M.-A.S.); lucanunzio.francioso@cnr.it (L.F.); 3 Pulmonology Scientific Research Institute, Federal Medical Biological Agency of Russia, Moscow 115682, Russia; kirillaz@inbox.ru (K.Z.); serpoff@mail.ru (V.B.); 4 Federal Center of Brain Research and Neurotechnologies, Federal Medical Biological Agency of Russia, Moscow 117513, Russia

**Keywords:** acoustic plate mode, silicon plate, piezoelectric film, liquid viscosity

## Abstract

The acoustic waves of higher orders propagating in a layered structure consisting of a silicon plate coated with piezoelectric *ZnO* and/or *AlN* films were used for the development of a sensor with selective sensitivity to liquid viscosity *η* in the range of 1–1500 cP. In that range, this sensor possessed low sensitivity to liquid conductivity *σ* and temperature T in the ranges of 0–2 S/m and 0–55 °C, respectively. The amplitude responses insensitive to the temperature instead of the phase were used to provide the necessary selectivity. The sensor was based on a weak piezoactive acoustic wave of higher order. The volume of the probes sufficient for the measurements was about 100 μL. The characteristics of the sensors were optimized by varying the thicknesses of the structure layers, number of layers, wavelength, wave propagation direction, and the order of the acoustic waves. It was shown that in the case of the layered structure, it is possible to obtain practically the same selective sensitivity toward viscosity as for acoustic waves in pure ST, X quartz. The most appropriate waves for this purpose are quasi-longitudinal and Lamb waves of higher order with in-plane polarization. It was found that for various ranges of viscosity *η* = 1–20 cP, 20–100 cP, and 100–1500 cP, the maximum sensitivity of the appropriate wave is equal to 0.26 dB/cP, 0.087 dB/cP, and 0.013 dB/cP, respectively. The sensitivity of the waves under study toward the electric conductivity of the liquid is much less than the sensitivity to liquid viscosity. These two responses become comparable only for very small *η* < 2 cP. The waves investigated have shown no temperature responses in contact with air, but in the presence of liquid, they increase depending on liquid properties. The temperature dependence of liquid viscosity is measurable by the same sensors. The results obtained have shown the possibility of designing acoustic liquid viscosity sensors based on multilayered structures. The set of possible acoustic waves in layered structures possesses modified propagation characteristics (various polarization, phase velocities, electromechanical coupling coefficients, and attenuations). It allows choosing an optimal acoustic wave to detect liquid viscosity only.

## 1. Introduction

The development of fast, precise, highly sensitive, and reliable liquid viscosity sensors is very important for various medical applications, e.g., for the quick analysis of human blood during and/or after a coronavirus infection [1]. These devices may also be used for control of the coagulation properties of blood under the influence of drug therapy or other external influences [2,3], the properties of food [4], fuels and lubricants [5], and other liquids. The ultrasonic sensors based on various types of acoustic waves may be used for all these applications [6,7,8,9,10]. Such devices are based on the changes in the acoustic characteristics (velocity, attenuation) due to a liquid sample deposition on an acoustic propagation path [11].

Recently, the possibility of developing an acoustic sensor with high sensitivity to the liquid viscosity and low sensitivity to the electric conductivity and temperature of the liquid sample based on a single ST, X quartz plate has been shown [12]. In this case, it was possible to optimize such sensors only by wave order, wave frequency, wavelength, and plate thickness.

It is well known that layered structures composed of a plate with a thickness *H* of acoustic wavelength *λ* (*H* ~ *λ*) and one or two piezoelectric films with a thickness *h* much less than *λ* (*h* << *λ*) offer a large variety of acoustic properties [13,14,15,16,17,18,19]. Propagation of the waves in the structures may be changed by proper selection of the mode number n, plate thickness H, film thickness h, and material film combination [20]. Excitation of the waves in the multilayered structures may be accomplished by the larger amount of transducer configurations than in uncoated piezoelectric plates [15,16]. Therefore, the plates coated with one or two films of the same or different materials as well as the same or different thicknesses may turn out to be attractive for liquid sensors.

There are known theoretical works devoted to the investigation of liquid viscosity on the properties of shear mode in a multilayered flexural bulk acoustic wave resonator (FBAR) [21], Love-type waves in a composite structure [22], a shear-horizontal surface acoustic wave (SH-SAW) in layered magnetoelectric structures [23], a surface acoustic wave (SAW) in multilayered structure *ZnO*/*SiO_2_*/*Si* [24], Love acoustic waves [25], quasi-Lamb modes in structure *SiC*/c-*ZnO* [26], etc. As it is well known, there have been attempts to create liquid acoustic sensors based on SAW [27], FBAR [28], and slot acoustic waves [29].

In ultrasonic sensors based on delay lines, a liquid sample is deposited on a plate between the input and output interdigital transducers (IDTs) and analyzed by an acoustic wave of various types (surface and plate waves with shear-horizontal polarization, antisymmetric and symmetric Lamb waves). Such a wave, depending on the frequency, penetrates into the liquid to a depth of 100–3000 nm [30,31]. The amplitude and phase of the acoustic wave are dependent on all liquid parameters together: that is, on density ρ, viscosity *η*, electrical conductivity *σ*, temperature *T* and permittivity ε [32,33,34]. Because of that, in order to define a set of liquid properties, it is possible to use several waves, multichannel sensor configurations, and special signal processing [35]. This peculiarity brings up the question: are there acoustic waves possessing simultaneously (i) sufficient electromechanical coupling coefficient to be excited, (ii) dominant sensitivity to liquid viscosity to be selective, and (iii) is it possible to develop a single channel sensor that is selectively sensitive to viscosity using an appropriate acoustic wave and relevant solid state structure?

The goal of the present paper is to study these questions using non-piezoelectric Si plates coated with piezoelectric (*ZnO*, *AlN*) films and changing wave properties through changing the propagation direction, plate/film or film/plate/film material combination, mode order *n*, plate thickness *H*, films thickness *h*, and acoustic wavelength *λ*.

## 2. Materials and Methods

### 2.1. Theoretical Methods

**Figure 1:**
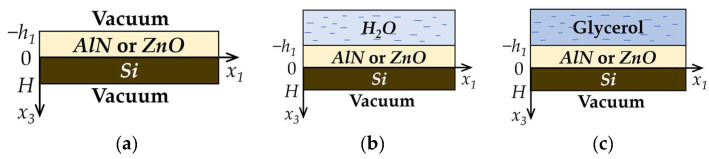
Geometries of the two-layered structure problems. Structure (**a**) without loading, (**b**) loaded by distilled water, (**c**) loaded by glycerol.

**Figure 2:**
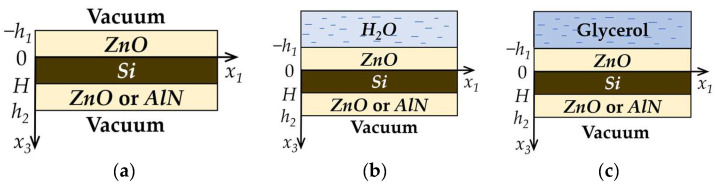
Geometries of the three-layered structure problems. Structure (**a**) without loading, (**b**) loaded by distilled water, (**c**) loaded by glycerol.

Acoustic waves propagate along the x_1_ direction of the piezoelectric structures consisting of one or two piezoelectric films (*ZnO* or *AlN*) deposited on the surface of the non-piezoelectric plate (*Si*). The geometries of the problems are shown in Figure 1 and Figure 2.

The non-piezoelectric plate is bounded by planes *x*_3_ = 0 and *x*_3_ = *H*. In a two-layered structure, a piezoelectric film (*ZnO* or *AlN*) is bounded by planes *x*_3_ = 0 and *x*_3_ = −*h*_1_ (Figure 1). In the case of a three-layered structure, one piezoelectric film (*ZnO*) is bounded by planes *x*_3_ = 0 and *x*_3_ = −*h*_1_, and the other one (*ZnO* or *AlN*) is bounded by planes *x*_3_ = *H* and *x*_3_ = *h*_2_ (Figure 2).

The regions *x*_3_ < −*h_1_* and *x*_3_ > *H* (Figure 1a), *x*_3_ < −*h_1_* and *x*_3_ > *h_2_* (Figure 2a) correspond to a vacuum. The task considered is a 2D problem, so all field components are assumed to be constant in the x_2_ direction [36]. The equation of motion, Laplace’s equation, and constitutive equations were used for each layer to find the phase velocity and mechanical displacements of the wave [37].

(1) $$\rho^{pz}\partial^{2}U_{i}^{pz}/\partial t^{2}=\partial T_{ij}^{pz}/\partial x_{j},\partial D_{j}^{pz}/\partial x_{j}=0,$$

(2) $$T_{ij}^{pz}=C_{ijkl}^{pz}\partial U_{l}^{pz}/\partial x_{k}+e_{kij}^{pz}\partial \Phi^{pz}/\partial x_{k},$$

(3) $$D_{j}^{pz}=-\varepsilon_{jk}^{pz}\partial \Phi^{pz}/\partial x_{k}+e_{jlk}^{pz}\partial U_{l}^{pz}/\partial x_{k},$$

(4) $$\rho^{npz}\partial^{2}U_{i}^{npz}/\partial t^{2}=\partial T_{ij}^{npz}/\partial x_{j},$$

(5) $$\partial D_{j}^{npz}/\partial x_{j}=0,$$

(6) $$T_{ij}^{npz}=C_{ijkl}^{npz}\partial U_{l}^{npz}/\partial x_{k},$$

(7) $$D_{j}^{npz}=-\varepsilon_{jk}^{npz}\partial \Phi^{npz}/\partial x_{k},$$

Here, *U_i_* is the component of the mechanical displacement of particles, *t* is the time, *T_ij_* is the component of the mechanical stress, *x_j_* is the coordinate, *D_j_* is the component of the electrical displacement, *Φ* is the electrical potential, ρ is the density, and *C_ijkl_*, *e_ikl_*, and *ε_jk_* are the elastic, piezoelectric, and dielectric constants, respectively. The indexes *pz* and *npz* denote that the corresponding variable refers to the piezoelectric film and non-piezoelectric layer, respectively.

In the regions of the vacuum, the electrical displacement is satisfied by Laplace’s equation:

(8) $$\partial D_{j}^{vac}/\partial x_{j}=0,$$

where $D_{j}^{vac}=-\varepsilon_{0}\partial \Phi^{vac}/\partial x_{j}$. Here, *ε_0_* is the vacuum permittivity, and index *vac* means that the variable refers to the vacuum.

Then, the relevant electric and mechanical conditions on each boundary for the two-layered structure (Figure 1a) are written as [37]:

(9) $$x_{3}=-h_{1}:\;T_{i3}^{pz}=0;\;\Phi^{pz}=\Phi^{vac};\;D_{3}^{pz}=D_{3}^{vac},$$

(10) $$x_{3}=0:\;U_{i}^{pz}=U_{i}^{npz};\;T_{i3}^{pz}=T_{i3}^{npz};\;\Phi^{pz}=\Phi^{npz};\;D_{3}^{pz}=D_{3}^{npz},$$

(11) $$x_{3}=H:\;T_{i3}^{npz}=0;\;\Phi^{npz}=\Phi^{vac};\;D_{3}^{npz}=D_{3}^{vac}.$$

Here, *i* = 1–3, *h*_1_ and *H* are the thicknesses of a piezoelectric film and non-piezoelectric plate, respectively.

In the case of the three-layered structure (Figure 2a), the boundary conditions (9), (10), (12), and (13) were used:

(12) $$x_{3}=H:\;U_{i}^{npz}=U_{i}^{pz};\;T_{i3}^{npz}=T_{i3}^{pz};\;\Phi^{npz}=\Phi^{pz};\;D_{3}^{npz}=D_{3}^{pz},$$

(13) $$x_{3}=h_{2}:\;T_{i3}^{pz}=0;\;\Phi^{pz}=\Phi^{vac};\;D_{3}^{pz}=D_{3}^{vac}.$$

In the case of the presence of semi-infinite non-viscous or viscous, non-conductive liquid on the structure surface in the region *x*_3_ < −*h*_1_ (Figure 1b and Figure 2b), it is necessary to use the equation of motion (4), Laplace’s Equation (5), and governing equations for $T_{ij}^{lq}$ (6) and $D_{j}^{lq}=-\varepsilon_{lq}\partial \Phi^{lq}/\partial x_{j}$ for liquid. In these equations, index *npz* should be replaced by *lq* one. The boundary conditions in the plane *x*_3_ = −*h*_1_ for this task should be written as follows [38]:

(14) $$x_{3}=-h_{1}:\;U_{3}^{lq}=U_{3}^{pz};\;T_{33}^{lq}=T_{33}^{pz};\;T_{13}^{pz}=T_{23}^{pz}=0;\;\Phi^{pz}=\Phi^{lq};\;D_{3}^{pz}=D_{3}^{lq}.$$

When in the region *x*_3_ < −*h*_1_ (Figure 1c and Figure 2c) the liquid is non-conductive but viscous, it is necessary to use the next boundary conditions [39]:

(15) $$x_{3}=-h_{1}:\;U_{i}^{pz}=U_{i}^{lq};\;T_{i3}^{pz}=T_{i3}^{lq};\;\Phi^{pz}=\Phi^{lq};\;D_{3}^{pz}=D_{3}^{lq}.$$

In this case, the nonzero components of symmetric complex elastic constants of viscous liquid $C_{ij}^{lq*}$ in matrix form will have the next form [39]:

(16) $$\begin{array}{c} C_{11}^{lq*}=C_{22}^{lq*}=C_{33}^{lq*}=C_{11}^{lq}+j\omega \eta_{11}^{lq}\\ C_{12}^{lq*}=C_{13}^{lq*}=C_{23}^{lq*}=C_{11}^{lq}+j\omega \eta_{12}^{lq}\\ C_{44}^{lq*}=C_{55}^{lq*}=C_{66}^{lq*}=j\omega \eta_{44}^{lq} \end{array},$$

where the viscosity of the liquid is accounted as an imaginary part $j\omega \eta_{11}^{lq}$ of elastic moduli, *j* is an imaginary value, ω = 2πf is the circular frequency, $\eta_{ij}^{lq}$ are viscosity coefficients in Pa×s, and $\eta_{12}^{lq}=\eta_{11}^{lq}-2\eta_{44}^{lq}$.

The values of wave phase velocity *v* and three partial components of mechanical displacement (*U*_1_, *U*_2_, *U*_3_) in the plane *x*_3_ = −*h*_1_ were found by using the method described in [38].

The solution is represented as a set of inhomogeneous waves for a set of equations for each medium (piezoelectric plate, non-piezoelectric plate, non-viscous or viscous liquid and vacuum):

(17) $$Y_{i}\left(x_{1},x_{3},t\right)=Y_{i}\left(x_{3}\right)e^{j\omega \left(t-x_{1}/v\right)}$$

Here, *Y_i_* represents normalized values of *U*_1_, *U*_2_, *U*_3_, *T*_13_, *T*_23_, *T*_33_, *Φ*, and *D*_3_ for each considered media [38].

This solution is substituted in appropriate equations corresponding to each medium of the considered structure. As a result, the systems of ordinary differential linear equations for each medium of structure are obtained. Each part of this system is represented in the matrix form as follows:

(18) $$\left[A\right]\left[{dY}_{i}/dx_{3}\right]=\left[B\right]\left[Y_{i}\right]$$

Here, [*dY_i_*/*dx_3_*] and [*Y_i_*] represent the vectors for the corresponding medium. The [*A*] and [*B*] are square matrices with dimensions of 8 × 8, 6 × 6, 4 × 4, 6 × 6, and 2 × 2 corresponding to a piezoelectric plate, a non-piezoelectric plate, non-viscous liquid, viscous liquid, and vacuum, respectively.

Since *det*[*A*] ≠ 0 for each contacting media, Equation (18) can be presented as follows:

(19) $$\left[{dY}_{i}/dx_{3}\right]=\left[A^{-1}\right]\left[B\right]\left[Y_{i}\right]=\left[C\right]\left[Y_{i}\right]$$

Then, the eigenvalues *c^(i)^* and corresponding eigenvectors $Y_{k}^{\left(i\right)}$ of matrix [*C*] for each medium were found. These values are determined the parameters of partial waves. The general solution is a linear combination of all partial waves for each medium.

(20) $$Y_{k}=\sum_{i=1}^{N}A_{i}Y_{k}^{\left(i\right)}e^{\left(c^{\left(i\right)}x_{3}\right)}e^{i\omega \left(t-x_{1}/v\right)}$$

Here, N = 8, 6, 4, 6, and 2 for a piezoelectric plate, a non-piezoelectric plate, non-viscous liquid, viscous liquid, and vacuum, respectively. *A_i_* represents the quantities unknown yet. The appropriate normalized electrical and boundary conditions (9)–(15) for each considered structure are used to define the quantities *A_i_* and wave phase velocity *v*. For semi-infinite media like vacuum and liquid, the eigenvalues and eigenvectors corresponding to the decreasing amplitude of variables with respect to the increasing distance from solids were taken into account. The so obtained matrix of the boundary conditions is dependent on *v*. Then, by using the descent method and iterative procedure with respect to the determinant of the boundary conditions matrix, the value of *v* is determined with certain accuracy (10^−7^). After that, the values of *A_i_* are determined to find *U*_1_, *U*_2_, and *U*_3_ in accordance with (20).

Material constants of *AlN*, *ZnO*, *Si*, distilled *H*_2_*O*, and glycerol are taken from [40,41,42] and presented in Table 1.

### 2.2. Experimental Methods

The measurements were carried out at room temperature (22.5 °C) and atmospheric pressure (743 mmHg). *Si* wafers with Euler angles 0°, 0°, 0° ((001), <100>—cut) and 45°, 54.7356°, 0° ((111), <110>—cut) were used as non-piezoelectric plates. The normalized thickness of the *Si* plates was in the range of *H*/*λ* = 0.625 to 2.6 (*H* = 250 and 380 μm, *λ* = 146, 200, and 400 μm). It allowed for providing a variety of acoustic modes suitable for the application. Piezoelectric *ZnO* and *AlN* films with the *c*-axis perpendicular to the surfaces (Euler angles 0°, 0°, 0°) are used as plate coatings allowing both wave generation and modification. The normalized thicknesses of *ZnO* and *AlN* films were in the range of *h_1,2_*/*λ* = 0.0005 to 0.084 (*h_1,2_* = 0.2 ÷ 12.3 μm, *λ* = 146, 200, and 400 μm).

The technology of the film’s fabrication accounted for the difference in the expansion coefficients of the *AlN*, *Si*, and *ZnO* materials. The *c*-oriented textured *AlN* was fabricated in a magnetron sputtering system using 50% *Ar* + 50% *N*_2_ gas mixture, 0.1 Pa gas pressure, and an *Al* target (99.999%) 140 mm in diameter. The distance between the target and substrate, the discharge power, the sputtering rate of the *AlN* film, and the substrate temperature were 70 mm, 800 W, 0.7–0.8 μm/h, and 150 °C, respectively [16].

The fabrication of the c-oriented *ZnO* films were performed in a triode sputtering system with a dc current using the *ZnO* target, 80% *Ar* + 20% *O_2_* gas mixture, and 0.07 Pa pressure. The substrate temperature was 200 °C, and the rate of the sputtering was 1.2–3 μm/h [16].

**Figure 3:**
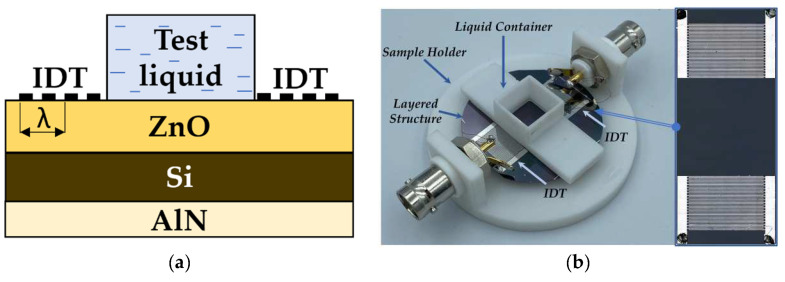
(**a**) Schematic view of a three-layered experimental structure; (**b**) Photo of the experimental delay line.

*ZnO* or *AlN* films were deposited on a *Si* plate from one or from both sides (Figure 3a). The input and output periodic interdigital transducers (IDTs) were deposited on the *ZnO* film for each structure used. Each transducer comprised 20 finger electrodes patterned from *Cr*(100 nm)/*Al*(1000 nm). The bandwidth of the transducers (5%) provides good frequency resolution of the neighboring modes with close velocities *v*. A liquid cell (fused quartz) was placed on the *ZnO* film surface between IDTs and glued to the surface by salol. The size of the liquid cell was large enough to avoid the perturbation of an acoustic beam by the cell walls. The photo of the produced delay line based on structure *ZnO*/*Si*/*AlN* as an example is presented in Figure 3b.

The mode velocities were measured as *v_exp_* = *λf*, where *λ* is the period of IDTs (the wavelength), and *f* is the central frequency of the modes of different types. The precision of the measurements was about ±1%.

The frequency dependencies of the insertion loss *S*_12_(*f*) were measured with precision ±0.1 dB by means of a KEYSIGHT 5061B network analyzer (Keysight, Santa Rosa, CA, USA) operating in an amplitude-frequency format. The amplitude-frequency format *S*_12_(*f*) was converted to the amplitude-time format *S*_12_(*τ*) in order to avoid the influence of electromagnetic leakage. For this purpose, the gate window was started just after the appearance of such leakage and stopped after the appearance of the useful acoustic signal. As the gate was on, the leakage was off and the time delay format *S*_12_(*τ*) converted back to the frequency format *S*_12_(*f*) without the electromagnetic leakage.

The procedure of the attenuation measurements was next. Initially, the frequency dependence of the insertion loss $S_{12}^{air}$(*f*) was measured for the delay line without liquid in a container. Then, the same frequency dependencies $S_{12}^{H_{2}O}(f)$, $S_{12}^{H_{2}O+Glycerol}(f)$, and $S_{12}^{Glycerol}(f)$ were measured in the presence of distilled *H_2_O* (*η* = 1.003 cP), *H_2_O* solutions of glycerol (1.003 cP < *η* < 1.491 cP) and pure glycerol (*η* = 1.491 cP), respectively. It allowed varying the viscosity of a test liquid in 3 orders of value, density of less than 26% and permittivity of less than 10.5% [38]. These liquids were placed into the cell one by one. After each use, the cell was cleaned and dried. The electric conductivity for all liquids used was equal to zero. After that, for each mode and test liquid, the attenuation coefficient *α* was determined as ${\alpha_{}=(S}_{12}^{Glycerol}-S_{12}^{H_{2}O})$/*L* = $S_{12}^{Glycerol-H_{2}O}/L$, where *L* is the propagation distance of the acoustic wave along the liquid/structure interface. Finally, for each liquid sample, the modes with the largest *α* were determined and compared with the largest values for other samples. Precisions of the measurements were ±0.01 dB and ±0.005 dB/mm for *S*_12_ and *α*, respectively.

**Figure 4:**
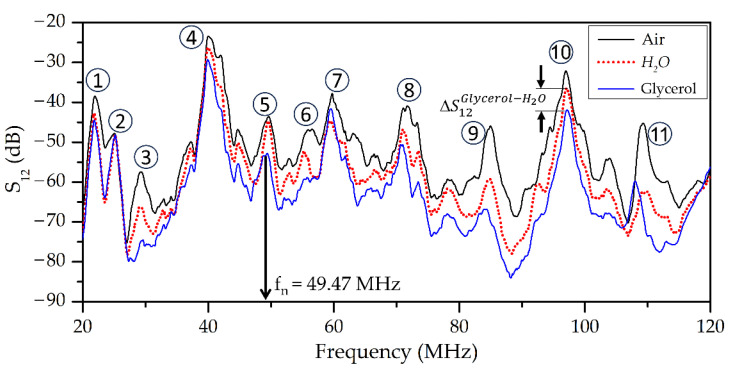
Typical frequency dependence of insertion loss *S_12_* for acoustic waves of different types propagating in a layered structure *ZnO* (*h*_1_ = 6.5 μm)/(111)*Si* (*H* = 390 μm)/*AlN* (*h*_2_ = 1.2 μm) without liquid (black line), in the presence of non-viscous, non-conductive water (red dashed line), and glycerol (blue line) placed on the ZnO film. The distance between IDTs L is equal to 16 mm and λ = 200 μm. The numbers from 1 to 11 indicate the serial number of the corresponding acoustic response through experiment.

The typical frequency dependencies of insertion loss *S_12_* of an acoustic wave family propagating in a layered structure *ZnO*/(111)*Si*/*AlN* without liquid (black line), in the presence of non-viscous, non-conductive water (red dashed line), and glycerol (blue line) placed on the *ZnO* film, are presented in Figure 4.

The water solutions of *NaCl* with electrical conductivity *σ* varied in the range of 0–10 S/m (7.6 weight % *NaCl* in *H_2_O*) were used to estimate the cross-sensitivity of the acoustic waves toward *σ*. A change in the conductivity within these limits left the viscosity (<13%), density (<8%), and permittivity (<1%) of the liquid almost constant [36].

Because the liquid properties have a strong dependence on temperature T, most of the measurements were carried out at 20 ± 0.1 °C fixed by a thermal camera UC-20CE (NOSELAB ATS, Nova Milanese, Italy). An experimental sample was mounted onto the camera and connected with the network analyzer in order to measure the insertion loss *S_12_* at relevant temperature. The camera with the sample was heated in the range of 0–55 °C with the step 5 °C and stopped for 5 min at each temperature to study the temperature sensitivity of the sensor developed. Without liquid, the sensor responded to temperature according to the properties of the wave whose amplitude is almost temperature independent (*S_12_* = const). In the presence of liquid, the amplitude response of the sensor behaves according to the liquid properties and *S_12_* = *S_12_* (*η*, *T*). The acoustic response Δ*S_12_* was preliminary calibrated toward viscosity using 9 samples of water solutions of glycerin with the known values of the viscosities [41]. So, when a liquid with unknown viscosity was used, the response Δ*S_12_* for this liquid was measured properly, and the relevant viscosity was determined directly from the aforementioned calibration curve. After such preliminary calibration toward viscosity η at a fixed temperature and extracting the 1st data from the 2nd, a liquid viscosity at relevant temperature T is measured as the difference. The precision of the measurement is Δ*S_12_* = ±0.2 dB. Changing the temperature *T* and following the same procedure, the temperature dependence of liquid viscosity *η*(*T*) was determined.

The sensitivity of acoustic waves toward liquid viscosity η at a fixed temperature is different in various *η* ranges [31]. Accordantly, precision of the measurements is also varied with *η*: for Δ*S_12_* measured at a fixed temperature with precision of ±0.1 dB, the accuracy of the η measurements is estimated as Δη/*η* ≈ 2.5% for *η* = 1–20 cP, ±5% for *η* =20–100 cP, and ±10% for *η* =100–1500 cP. On the other hand, when viscosity *η* is determined versus temperature *T*, the difference Δ*S_12_* is measured for two different temperatures with precision ±0.1 dB for each and ±0.2 dB for two. As a result, the accuracy of the *η*(*T*) measurements drops twice, becoming ±5% for *η* = 1–20 cP, ±10% for *η* =20–100 cP, and ±20% for *η* =100–1500 cP.

The mix of partial components in a forced vibrator for about 5 min is allowed to obtain liquid solutions used in experiments. By using data from [41] and knowing the weights of each component, the values of *η* and *σ* for liquid solutions at 20 °C were determined. An error in the weight concentration of glycerol and *NaCl* in water was less than ±1%. The volume of the test liquids used for the measurements was 100 μL. 

The surface amplitudes of the longitudinal *U*_1_, shear-horizontal *U*_2_ and shear-vertical *U_3_* mode displacements calculated theoretically were used to study the correlation between acoustic attenuation *α* on one hand and polarization of the waves on the other hand.

## 3. Results and Discussion

Nine experimental delay lines based on various structures were considered during investigations. Most of the examined waves are not useful for application due to large attenuation in the presence of liquid. The reasons for such attenuation are compressional wave radiation into liquid due to the presence of the surface-normal wave component u_3_ and visco-elastic coupling of the modes and liquid due to in-plane components u_1_ and u_2_. However, some waves with allowable attenuation *α_n_* were found even though the insertion loss of the sensor loaded with glycerol approaches 94 dB (Table 2). Glycerol responses of the best waves referred to that of water $\alpha_{}^{Glycerol-H_{2}O}\;$were as large as 0.8–1.6 dB/mm. These values are marked as a bold in Table 2. It has been found that using an additional piezoelectric layer over the second side of the *Si* plate leads to a change in acoustical response $\alpha_{}^{Glycerol-H_{2}O}$ (Table 2, lines 4 and 5). So, it is possible to control the acoustic wave properties by a piezoelectric film placed on the surface of a non-piezoelectric plate free of IDTs.

**Figure 5:**
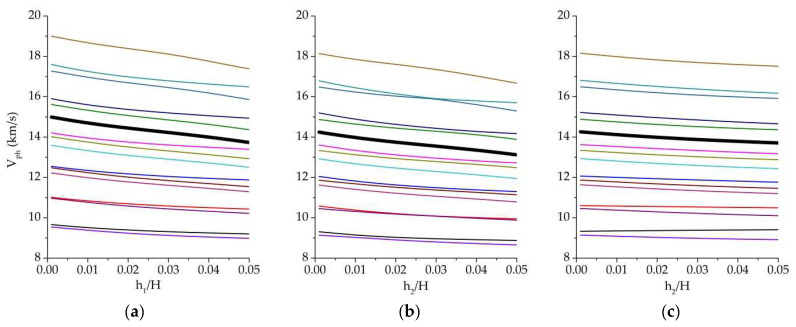
Typical dependence of phase velocity of acoustic waves of different types propagating in layered structures vs. thickness of piezoelectric film: (**a**) *ZnO* (*h*_1_ = 0.38 ÷ 19 μm)/(100)*Si* (*H* = 380 μm), (**b**) *ZnO* (*h*_1_ = 10.5 μm)/(100)*Si* (*H* = 380 μm)/*ZnO* (*h*_2_ = 0.38 ÷ 19 μm), (**c**) *ZnO* (*h*_1_ = 10.5 μm)/(100)*Si* (*H* = 380 μm)/*AlN* (*h*_2_ = 0.38 ÷ 19 μm) without liquid. Bold black line corresponds to wave #7 from Table 3. Lines of various colors are corresponded to acoustic waves of different types and order number (symmetric, antisymmetric and shear-horizontal).

Theoretical analysis of the acoustic waves of higher orders propagating in such experimental structures was performed using Equations (1)–(16). As the results of numerical calculations, the dispersion curves for all considered structures were plotted. In Figure 5, the typical dependencies of the phase velocity of acoustic waves of various types vs. the thickness of corresponding piezoelectric film/films are presented.

It has been found that the increase in piezoelectric film thickness leads to a decrease in the phase velocities of acoustic waves of various types in *Si* plate. The *ZnO* film placed on the other side of the *Si* plate (Figure 5b) leads to a greater change of phase velocity of acoustic waves than *AlN* film (Figure 5c).

The results of the calculation of the phase velocities, attenuation, and mechanical displacements in the plane *x*_3_ = −*h*_1_ are presented in Table 3. The comparison of the theoretical and experimental results has shown good agreement. It has been theoretically confirmed that using two piezoelectric films placed on both sides of the *Si* plate allowed controlling the properties of an acoustic wave.

**Figure 6:**
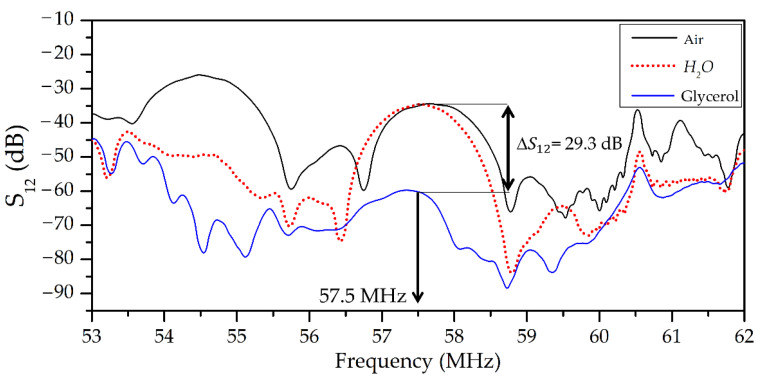
Frequency dependencies of insertion loss *S_12_* of wave #9, Table 2 measured in the air, with water, or with glycerol loading. Polarization of the mode in the plane *x*_3_ = −*h*_1_ is quasi-longitudinal: *v_th_* = 8365 m/s, *U*_1_ = 1; *U*_2_ = 0; *U*_3_ = 0.012.

**Figure 7:**
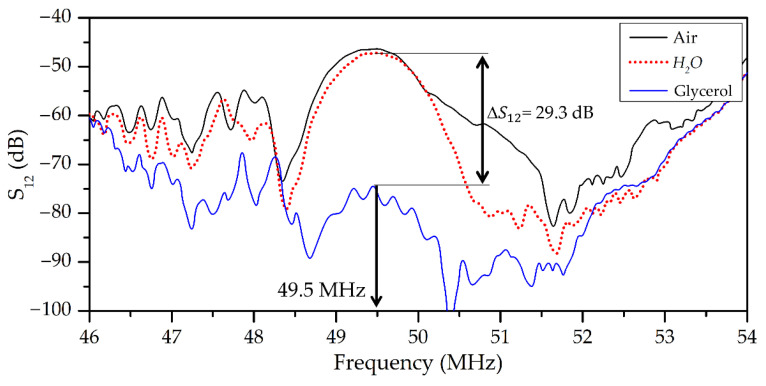
Frequency dependencies of insertion loss *S_12_* of wave #4, Table 2 measured in the air, with water, or with glycerol loading. Polarization of the mode in the plane *x*_3_ = −*h*_1_ is elliptic with displacement ellipse almost parallel to the plate faces: *v_th_* = 9781 m/s, *U*_1_ = 1; *U*_2_ = 1.1; *U*_3_ = 0.1.

Examples of the frequency dependencies of insertion loss *S*_12_ for the wave with dominant longitudinal displacement (*U*_1_ >> *U*_2_, *U*_3_; *f* = 57.5 MHz) and wave with elliptic in-plane polarization (*U*_1_ ≈ *U*_2_, *U*_3_ ≈ 0; *f* = 49.5 MHz) are presented in Figure 6 and Figure 7, respectively. These waves have a negligible surface-normal component *U_3_*. This feature is eliminated radiation of compressional wave into adjacent liquid. It is possible to see that the measured *S_12_* for both waves are almost the same with water loading and in the air ($S_{12}^{H_{2}O}$ = $S_{12}^{Air}$). On the other hand, these waves have large in-plane components *U*_1_ for *f* = 57.5 MHz and *U*_1,_ *U*_2_ for *f* = 49.5 MHz. So, they are strongly coupled with adjacent liquid and possess high sensitivity to liquid viscosity, and they are suited for viscosity measurements. It is necessary to note that other waves of the same structure at *f* = 54.48 MHz (Figure 6) have remarkable normal components (*U*_3_ = 0.51*U*_1_) and, thereby, are characterized by big radiation loss into the liquid sample (>25 dB). Due to this, such a wave is damped by any liquid and is not suited for viscosity measurement application.

**Figure 8:**
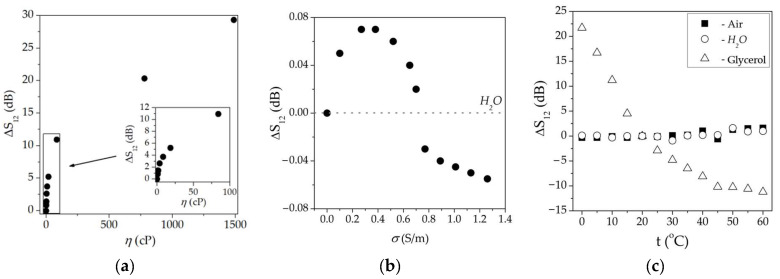
Calibration curves of the sensor based on mode #4, Table 2 toward (**a**) viscosity of water solution of glycerol, (**b**) electric conductivity of water solution of *NaCl*, and (**c**) temperature. The frequency of the mode is 49.5 MHz.

The liquid viscosity sensor prototypes were realized for all appropriate waves. For example, in Figure 8, the dependencies of the change in the insertion loss Δ*S_12_* versus viscosity, conductivity, and temperature of liquid measured through the sensor based on wave #4 (Table 2) are presented. The concentration curve for liquid viscosity (Figure 8a) is almost linear for *η* in the range of 0–20 cP when the liquid is Newtonian. At *η*, about 1500 cP the liquid starts to behave as a solid and Δ*S_12_* approaches saturation [7]. As a result, the sensitivity of the sensor toward viscosity is varied from 0.26 dB/cP for *η* = 1–20 cP to 0.087 dB/cP for *η* = 20–100 cP and 0.013 dB/cP for *η* = 100–1500 cP.

The dependence of change in insertion loss Δ*S*_12_ versus liquid conductivity has a typical view [38] (Figure 8b). It increases for small *σ* < 0.4 S/m, approaches maximum at σ = 0.4 S/m, and falls to zero for large σ > 1 S/m. These measurements were performed to estimate the cross-sensitivity of the acoustic waves under study toward liquid conductivity. It is possible to see that in the case of using waves in multilayered structures instead of quartz plates [12], the sensitivity toward liquid conductivity is less for these waves (0.08 dB < 0.2 dB). It could be explained by the smaller value of the electromechanical coupling coefficient of the waves in multilayered structures than in quartz plates. As a result, it is possible to see that the electric responses of the acoustic wave (Figure 8b) are much less than those for viscosity (Figure 8a). Only for very small *η* < 2 cP do these two responses become comparable.

The temperature sensitivity of the sensor based on two-layered and three-layered structures without liquid loading and loading with non-viscous, non-conductive *H_2_O* is negligible (Figure 8c).

**Figure 9:**
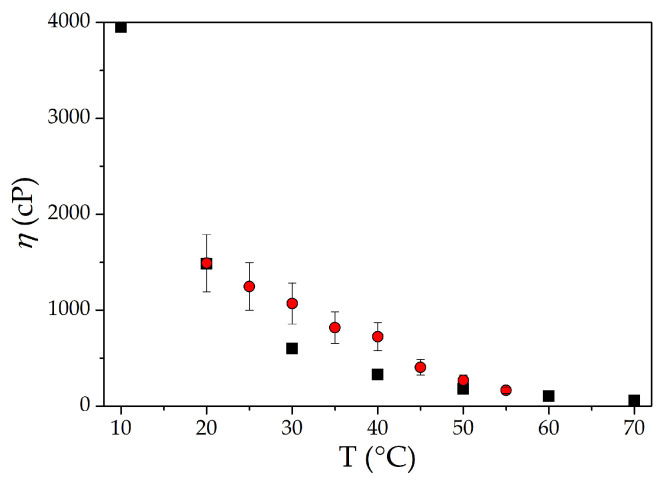
The temperature dependence of glycerol viscosity measured in this paper (red rings) by using mode #4 from Table 2 with f = 49.5 MHz and the same dependence taken from [41] (black squares).

However, in the presence of a viscous liquid, the sensor responds to the temperature accordingly. It is possible to extract the data for unloaded structures from the data for structures loaded by viscous liquid at various temperatures. As a result, we obtained the temperature dependence of viscosity *η*(*T*) (red rings) for glycerol (Figure 9). The comparison of the obtained results with the tabulated (black squares [41]) ones has shown good agreement.

## 4. Conclusions

Acoustic waves with increased sensitivity to liquid viscosity and decreased sensitivity to liquid conductivity are found in layered structures composed of the non-piezoelectric *Si* plates and piezoelectric *ZnO* (*AlN*) films. The waves belong to quasi-longitudinal and Lamb wave families with polarization parallel to plate faces. Small surface-normal displacement avoids the wave radiation into adjacent liquid. Large in-plane components enhance the viscous coupling the waves and liquids deposited on the plate.

The sensors based on these waves and selectively sensitive to liquid viscosity are developed. The sensors have an amplitude output with sensitivities 0.26 dB/cP for *η* = 1–20 cP, 0.087 dB/cP for *η* = 20–100 cP, and 0.013 dB/cP for *η* = 100–1500 cP. Responses of the sensor toward liquid conductivity (0 to 2 S/m) are two orders of value smaller, becoming comparable with those for viscosity only at *η* < 2 cP. The temperature responses of the sensors are almost zero in air, but when coated with liquid, they increase depending on the liquid properties. The dependence of liquid viscosity versus temperature is measurable by the same sensor.

As compared with the reference structure based on an uncoated quartz plate [12], the coated prototypes developed in this paper have the same sensitivity toward viscosity and temperature, but they have less sensitivity toward liquid conductivity (0.12 dB vs. 0.2 dB for *σ* = 0 – 1.4 S/m). Also, the glycerin response Δ*S_12_* of the best mode in the coated sensor is higher than that in the uncoated prototype (29.3 dB vs. 27 dB).

The results obtained have shown the possibility of designing acoustic liquid viscosity sensors based on multilayered structures. The set of possible waves with various polarization, phase velocities, electromechanical coupling coefficients, and attenuations expands the possibilities of developing acoustic sensors with selective response only to liquid viscosity.

## Figures and Tables

**Table 1:** Density *ρ* (kg/m^3^), elastic constants *C_ij_* (GPa), piezoelectric coefficients *e_ij_* (C/m^2^), viscosity coefficients *η_ij_* (Pa × s) and dielectric permittivity *ε_ij_*/*ε_0_* of *AlN*, *ZnO*, *Si*, distilled *H_2_O* and glycerol used in calculations (T = 22.5 C).

*AlN* (*ρ* = 3260)										
*C^E^*_11_	*C^E^*_12_	*C^E^*_13_	*C^E^*_33_	*C^E^*_44_	*C^E^*_66_	*e*_15_	*e*_31_	*e*_33_	*ε*_11_/*ε*_0_	*ε*_33_/*ε*_0_
345	125	120	395	118	110	−0.48	−0.58	1.55	8	9.5
*ZnO* (*ρ* = 5665)										
*C^E^*_11_	*C^E^*_12_	*C^E^*_13_	*C^E^*_33_	*C^E^*_44_	*C^E^*_66_	*e*_15_	*e*_31_	*e*_33_	*ε*_11_/*ε*_0_	*ε*_33_/*ε*_0_
209	120.5	104.6	210.6	42.3	44.55	−0.48	−0.573	1.321	7.57	9.03
*Si* (*ρ* = 2330)				*H_2_O* (*ρ* = 997.299)		*Glycerol* (*ρ* = 1260)				
*C^E^*_11_	*C^E^*_12_	*C^E^*_44_	*ε*_11_/ε_0_	*C*_11_	*ε*/*ε*_0_	*C*_11_	*η*_11_	*C*_44_	*η*_44_	*ε*/*ε*_0_
166	63.9	79.6	10.62	2.25	80	2.81	118.6	1.2128 × 10^−3^	1.5	41.9

**Table 2:** Acoustic plate waves with the largest sensitivity toward glycerol measured in different samples at 20 °C. The thicknesses of the plate and films are presented in brackets. *v_exp_* is the wave velocity in the structure without liquid loading.

No.	Layers (Thickness) (μm)/(μm), Frequency	H/λ	λ, μm	L, mm	*v_exp_*, m/s	$S_{12}^{Glycerol}$, dB	$S_{12}^{Glycerol-H_{2}O},$ dB	${∆\alpha }_{}^{Glycerol-H_{2}O}$, dB/mm
1	*IDT/AlN*(1.8)/(111)Si (250) *f* = 67.94 MHz	0.625	400	45	27,200	94 ± 0.1	8 ± 0.1	0.18 ± 0.002
2	*IDT/*ZnO(0.4)/(100)Si (380)/ZnO(10) *f* = 34.6 MHz	1.9	200	16	13,900	58 ± 0.1	7 ± 0.1	0.44 ± 0.006
3	*IDT/*ZnO(10)/(100) Si(380) *f* = 43.46 MHz	1.9	200	16	8700	67 ± 0.1	12.7 ± 0.1	0.8 ± 0.006
4	*IDT/*ZnO(6.3)/(111) Si(380) *f* = 49.5 MHz	1.9	200	37	9900	**75** ± 0.1	**29.3** ± 0.1	**0.8** ± 0.003
5	*IDT/*ZnO(6.5)/(111)Si(380)/AlN(1.2) *f* = 49.5 MHz	1.9	200	16	9900	53 ± 0.1	8 ± 0.1	0.5 ± 0.006
6	*IDT/*ZnO(10)/(100)Si(380)/AlN(0.2) *f* = 48.8 MHz	1.9	200	16	9800	66 ± 0.1	8 ± 0.1	0.5 ± 0.006
7	*IDT/*ZnO(10.5)/(100)Si(380) *f* = 72.75 MHz	1.9	200	16	14,600	**68** ± 0.1	**17.5** ± 0.1	**1.1** ± 0.006
8	*IDT/*ZnO(10.5)/(100)Si(380) *f* = 74.76 MHz	1.9	200	16	15,000	**77** ± 0.1	**22.2** ± 0.1	**1.4** ± 0.006
9	*IDT/*ZnO(8.8)/(100)Si(380)/ZnO(5.6) *f* = 57.5 MHz	2.6	146	16	8400	**61** ± 0.1	**25** ± 0.1	**1.6** ± 0.006
10	*IDT/*ZnO(8.8)/(100)Si(380)/ZnO(5.6) *f* = 51.24 MHz	2.6	146	16	7500	**56** ± 0.1	**18** ± 0.1	**1.13** ± 0.006
11	*IDT/*ZnO(12.3)/(111)Si (380) *f* = 32.1 MHz	1.9	200	37	6400	57 ± 0.1	18.3 ± 0.1	0.5 ± 0.003

**Table 3:** The theoretically obtained phase velocities *v_th_*, normalized displacements U_1_, U_2_, and U_3_ at *x*_3_ = −*h*_1_, and attenuation coefficients α of the higher-order acoustic waves in two- and three-layered structures in the air, in the presence of non-viscous, non-conductive *H*_2_*O* or glycerol placed on the *ZnO* film (*x*_3_ < −*h*_1_).

No.	Layers (Thickness) (μm)/(μm), Frequency	*H*/*λ*	*λ*, μm	*L*, mm	Free Structure *v_th_*, m/s (*U*_1_; *U*_2_; *U*_3_) α, dB/mm	Structure + *H*_2_*O* *v_th_*, m/s (*U*_1_; *U*_2_; *U*_3_) α, dB/mm	Structure + Glycerol *v_th_*, m/s (*U*_1_; *U*_2_; *U*_3_) α, dB/mm
1	*AlN*(1.8)/(111)Si (250) *f* = 67.94 MHz	0.625	400	45	25,515	25,514	25,485
					(1; 0.45; 0.42)	(1; 0.4; 0.3)	(1; 0.45; 0.6)
					0	0	0.1
2	ZnO(0.4)/(100)Si (380)/ZnO(10) *f* = 34.6 MHz	1.9	200	16	13,937	13,936	13,917
					(1; 0; 0.73)	(1; 0; 0.3)	(1; 0; 0.8)
					0	0.1	0.1
3	ZnO(10)/(100) Si(380) *f* = 43.46 MHz	1.9	200	16	8438	8437	8415
					(1; 0; 2.86)	(1; 0; 2)	(1; 0; 2.2)
					0	0	0.1
4	ZnO(6.3)/(111) Si(380) *f* = 49.5 MHz	1.9	200	37	9781	9780	9767
					(1; 1.1; 0.1)	(1; 0.9; 0)	(1; 1.1; 0.1)
					0	0	0.5
5	ZnO(6.5)/(111)Si(380)/AlN(1.2) *f* = 49.5 MHz	1.9	200	16	9894	9893	9876
					(1; 0.82; 0.22)	(1; 0.6; 0.1)	(1; 0.8; 0.2)
					0	0	0.1
6	ZnO(10)/(100)Si(380)/AlN(0.2) *f* = 48.8 MHz	1.9	200	16	9342	9341	9312
					(1; 0; 0.09)	(1; 0; 0.01)	(1; 0; 0.1)
					0	0	0.1
7	ZnO(10.5)/(100)Si(380) *f* = 72.75 MHz	1.9	200	16	14,278	14,277	14,253
					(1; 6140; 1.1)	(1; 120; 0.05)	(1; 130; 0.4)
					0	0	0.55
8	ZnO(10.5)/(100)Si(380) *f* = 74.76 MHz	1.9	200	16	15,629	15,628	15,589
					(1; 14120; 1.8)	(1; 1500; 1.)	(1; 134; 1.2)
					0	0	0.6
9	ZnO(8.8)/(100)Si(380)/ZnO(5.6) *f* = 57.5 MHz	2.6	146	16	8365	8364	8341
					(1; 0; 0.012)	(1; 0; 0.002)	(1; 0; 0.015)
					0	0	0.55
10	ZnO(8.8)/(100)Si(380)/ZnO(5.6) *f* = 51.24 MHz	2.6	146	16	7382	7381	7364
					(1; 0; 0.7)	(1; 0; 0.5)	(1; 0; 0.75)
					0	0.2	0.45
11	ZnO(12.3)/(111)Si (380) *f* = 32.1 MHz	1.9	200	37	6425	6424	6395
					(1; 2; 0.48)	(1; 1.7; 0.2)	(1; 2.2; 0.3)
					0	0	0.1

## Data Availability

No new data were created or analyzed in this study. Data sharing is not applicable to this article.
